# ﻿*Artemisiaqingheensis* (Asteraceae, Anthemideae), a new species from Xinjiang, China

**DOI:** 10.3897/phytokeys.229.101689

**Published:** 2023-08-02

**Authors:** Guang-Zhao Jin, Mariya Sheludyakova, Wen-Jun Li, Feng Song, Zhi-Bin Wen, Ying Feng

**Affiliations:** 1 State Key Laboratory of Desert and Oasis Ecology, Xinjiang Institute of Ecology and Geography, Chinese Academy of Sciences, Urumqi 830011, China Xinjiang Institute of Ecology and Geography, Chinese Academy of Sciences Urumqi China; 2 The Herbarium of Xinjiang Institute of Ecology and Geography, Chinese Academy of Sciences, Urumqi 830011, China The Herbarium of Xinjiang Institute of Ecology and Geography, Chinese Academy of Sciences Urumqi China; 3 University of Chinese Academy of Sciences, Beijing 100094, China University of Chinese Academy of Sciences Beijing China; 4 Komarov Botanical Institute of Russian Academy of Sciences, Prof. Popova 2, Sankt Peterburg 197376, Russia Komarov Botanical Institute of Russian Academy of Sciences Sankt Peterburg Russia; 5 Key Laboratory of Plant Resources Conservation and Sustainable Utilization, South China Botanical Garden, Chinese Academy of Sciences, Guangzhou 510650, China South China Botanical Garden, Chinese Academy of Sciences Guangzhou China

**Keywords:** *Artemisia* subg. *Seriphidium*, Compositae, new taxon, taxonomy, Xinjiang

## Abstract

*Artemisiaqingheensis* (Asteraceae, Anthemideae), a new species from Qinghe County, Xinjiang, China, is described and illustrated. We investigated its phylogenetic position and relationships with 35 other species of *Artemisia* using whole chloroplast DNA sequence data. The molecular phylogenetic results and morphological evidence (multi-layered involucral bracts and homogamous capitula with bisexual flowers) showed that the new species belongs to ArtemisiasubgenusSeriphidium. A diagnostic table and discussion of morphological characters are provided to distinguish the new species from *A.amoena*, *A.gracilescens*, *A.lessingiana* and *A.terrae-albae*.

## ﻿Introduction

*Artemisia* L. (Asteraceae, Anthemideae), comprising ca. 500 herb and shrub species, is one of the largest genera in the tribe Anthemideae of the family Asteraceae (Bremer and Humphries 1993; Martin et al. 2003; Oberprieler et al. 2009; Vallès et al. 2011). Most *Artemisia* species have important medicinal, ecological and economic values (Duffy and Mutabingwa 2006; Vallès et al. 2011). Recent molecular phylogenetic studies of *Artemisia* have divided it into six subgenera, which are generally accepted: subg. Artemisia, subg. Absinthium (Miller) Less., subg. Dracunculus (Besser) Rydb., subg. Tridentatae (Rydb.) McArthur., subg. Seriphidium Besser ex Less and subg. Pacifica Hobbs & Baldwin (Malik et al. 2017, and references therein).

Subgenus Seriphidium, comprising ca. 130 species, is one of the most diverse subgenera and is mainly distinguished from the others by its multi-layered involucral bracts and homogamous capitula with bisexual flowers (Ling 1991). Subgenus Seriphidium grows mainly in arid and semi-arid regions in Central Asia and Northwest China (Malik et al. 2017). Thirty-one species and six varieties have been recorded in China (Ling et al. 2011).

During a field expedition in the north-eastern region of the Junggar Basin, located in Xinjiang, China, in 2020, a new population of *Artemisia* from Qinghe County was discovered. After consulting “Flora of China” (Ling et al. 2011) and other relevant literature (Poljakov 1961; Filatova 1966, 1986, 1993, 2007; Ling 1991; Liu 1992; Wei 1999), and after comparing the plants of this population with those of morphologically similar species (Besser 1841; Krascheninnikov 1930; Krascheninnikov and Iljin 1949; Poljakov 1954), we revisited this site at different times in 2021 and 2022 to carry out further observations and sampling with the aim of determining the taxonomic identity of the new population. Following additional morphological and molecular phylogenetic analyses, we concluded that it is different from all other known species of *Artemisia*. Hence, it is here described and illustrated as a new species: *A.qingheensis*.

## ﻿Material and method

After examining the worldwide list of subg. Seriphidium species and their type specimens (Jin 2023), we critically examined specimens (including type material) of *A.gracilescens* Krasch. & Iljin, *A.lessingiana* Besser, and *A.terrae-albae* Krasch. in IBSC, LE, LECB, MW, PE, TK, TASH and XJBI. These species are morphologically most similar to the new taxon.

**Figure 1.**
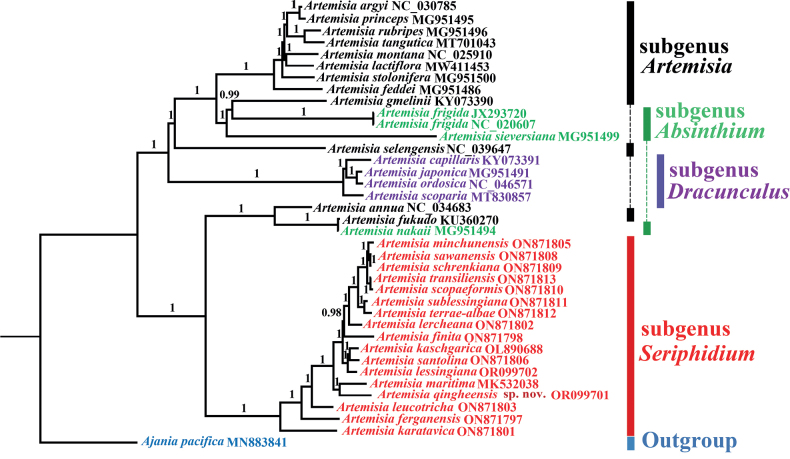
Phylogenetic tree inferred with Bayesian Inference (BI) analyses, using complete chloroplast genome sequences of 37 *Artemisia* species and *Ajaniapacifica* as the outgroup. The numbers above the branches are Bayesian posterior probabilities. Coloured vertical lines indicate the subgenus classification of *Artemisia*.

Chloroplast genomes of 36 *Artemisia* species from four subgenera, including 17 subg. Seriphidium species, were used for phylogenetic analysis (Fig. 1). The closely related species *Ajaniapacifica* (Table 1) was used as the outgroup (Watson et al. 2002). We included 38 samples in our phylogenetic analyses, 36 of them were obtained from NCBI (https://www.ncbi.nlm.nih.gov/) and two were newly sequenced for this study: *A.lessingiana* and *A.qingheensis* (Table 1). For both, we extracted total genomic DNA from approximately 100 mg of silica gel-dried leaf material using a modified CTAB method (Doyle and Doyle 1987). Voucher specimens (*A.qingheensis*: No. jgz-099-4; *A.lessingiana*: No. jgz-20220529) were deposited in the Herbarium of the Xinjiang Institute of Ecology and Geography Chinese Academy of Sciences (XJBI). DNA extracts were fragmented for short-insert library construction (300 bp) and sequenced (2 × 150 bp paired-end reads) on DNBSEQ technology platforms at the Beijing Genomics Institute (Shenzhen, China). The raw reads were assessed and edited using FastQC 0.11.5 (http://www.bioinformatics.babraham.ac.uk/projects/fastqc/) and Trimmomatic 0.35 (Bolger et al. 2014) was used to remove adapters and low quality bases. Finally, a ca. 3 G bp paired-end clean read was obtained for each sample. The clean data was assembled with GetOrganelle v. 1.7.1 (Jin et al. 2020). The complete circular assembly graph was checked using Bandage v. 0.8.1 (Wick et al. 2015). The finished plastid genomes were annotated with Geneious v. 9.1.7 (Kearse et al. 2012). The annotated plastid genomes were submitted to GenBank using Bankit (Table 1).

**Table 1.** Samples information. Highlighted species newly were sequenced in this study.

Species	GenBank No.	Species	GenBank No.
*Ajaniapacifica*	MN883841	*Artemisiaminchunensis*	ON871805
*Artemisiaannua*	NC_034683	*Artemisiamontana*	NC_025910
*Artemisiaargyi*	NC_030785	*Artemisianakaii*	MG951494
*Artemisiacapillaris*	KY073391	*Artemisiaordosica*	NC_046571
*Artemisiafeddei*	MG951486	*Artemisiaprinceps*	MG951495
*Artemisiaferganensis*	ON871797	***Artemisiaqingheensis*** sp. nov.	*OR099701*
*Artemisiafinita*	ON871798	*Artemisiarubripes*	MG951496
*Artemisiafrigida*	JX293720	*Artemisiasantolina*	ON871806
*Artemisiafrigida*	NC_020607	*Artemisiasawanensis*	ON871808
*Artemisiafukudo*	KU360270	*Artemisiaschrenkiana*	ON871809
*Artemisiagmelinii*	KY073390	*Artemisiascopaeformis*	ON871810
*Artemisiajaponica*	MG951491	*Artemisiascoparia*	MT830857
*Artemisiakaratavica*	ON871801	*Artemisiaselengensis*	NC_039647
*Artemisiakaschgarica*	OL890688	*Artemisiasieversiana*	MG951499
*Artemisialactiflora*	MW411453	*Artemisiastolonifera*	MG951500
*Artemisialercheana*	ON871802	*Artemisiasublessingiana*	ON871811
***Artemisialessingiana***	*OR099702*	*Artemisiatangutica*	MT701043
*Artemisialeucotricha*	ON871803	*Artemisiaterrae-albae*	ON871812
*Artemisiamaritima*	MK532038	*Artemisiatransiliensis*	ON871813

Genomes were aligned in MAFFT v. 7 (Katoh and Standley 2013). According to the Akaike Information Criterion (AIC), the most appropriate substitution model for the complete chloroplast genome sequence matrix, estimated using jModelTest2 (Darriba et al. 2012), was GTR + I + G. Bayesian Inference (BI) analysis was carried out using MrBayes v.3.2 (Ronquist et al. 2012), with the Markov Chain Monte Carlo simulations algorithm (MCMC) for 20,000,000 generations. The final trees were edited and visualised with FigTree v. 1.4.2 (Rambaut 2012).

## ﻿Results

The new species has multi-layered involucral bracts and homogamous capitula with bisexual flowers and therefore belongs to subg. Seriphidium. Its hardened needle-like leaves at maturity distinguish it from morphologically similar species: *A.gracilescens*, *A.lessingiana*, and *A.terrae-albae*. The results of the phylogenetic analyses showed that the new species is nested in a clade formed by subg. Seriphidium species (posterior probability (PP) = 1) and that it is the sister group (PP = 1) of *A.maritima* L. (Fig. 1). The new species is more distantly related to *A.lessingiana* and *A.terrae-albae*. In conclusion, the morphological characters and molecular data support the new species as distinct.

### ﻿Taxonomic treatment

#### ﻿ Artemisia qingheensis

Taxon classificationPlantaeAsteralesAsteraceae

G.Z.Jin sp. nov.

F6BAEE49-C971-5363-9450-99454DC0A980

urn:lsid:ipni.org:names:77324802-1

Figs 2A–M , 3

##### Type.

China. Xinjiang: Qinghe County, Qinglong Lake, 46°40'N, 90°23'E, barren slopes, 1168.63 m alt., 7 October 2021, *Guangzhao Jin & Lei Yang jgz-17* (holotype: XJBI jgz-17-2, Fig. 3; isotypes: XJBI jgz-17-1, jgz-17-3 and jgz-17-4).

##### Description.

Herbs perennial, 10–40 cm tall, with a thick rootstock, grey-white arachnoid pubescent, later glabrescent. Stems numerous, erect and often forming dense clumps, slightly woody proximally, herbaceous distally and with branches distally; branches 3–15 cm long, growing adnate to the stem, occasionally shorter branches. Lower stem leaves: petiole 0.3–1 cm; leaf blade elliptic, 0.5–1.5 cm long, 0.3–1 cm wide, 2-pinnatisect; primary segments 2–4 pairs; ultimate segments narrowly linear, 0.3–0.8 cm long and 0.2–0.5 mm wide, apex acute; petiole base with three-lobed or undivided pseudostipules with linear ultimate segments. Middle stem leaves: leaf blade narrowly ovate, 1 (or 2)-pinnatisect; ultimate segments narrowly linear, 0.5–1.5 cm long and 0.2–0.5 mm wide, apex acute; sessile, base with linear undivided pseudostipules. Upper leaves and leaf-like bracts: three-lobed or undivided, ultimate segments narrowly linear, 0.3–0.8 cm. All leaves greyish-white arachnoid pilose during the vegetative period, nearly glabrous at maturity; developing a needle-like texture at maturity. Inflorescence narrowly spicate or spicate-paniculate. Capitula sessile, numerous, ovoid, 2.5–4 mm long and 1.5–2.5 mm in diam., flowers opening centrifugally. Involucral bracts in 3–4 series, oblong or elliptic, 2–4 mm long and 1.5–2.5 mm wide, subglabrous, margin scarious; outer bracts ovate, inner larger, oblong-elliptic, all bracts with only sparse hairs at apex. Flowers bisexual, 3–6, 2–3.5 mm long and 1–2 mm wide, corolla tubular, purple-red or yellow; anthers linear, apical appendages of anthers subulate. Achenes with inconspicuous fine longitudinal lines, ovoid or obovoid, 1–1.5 mm long and 0.3–0.8 mm wide.

**Figure 2.**
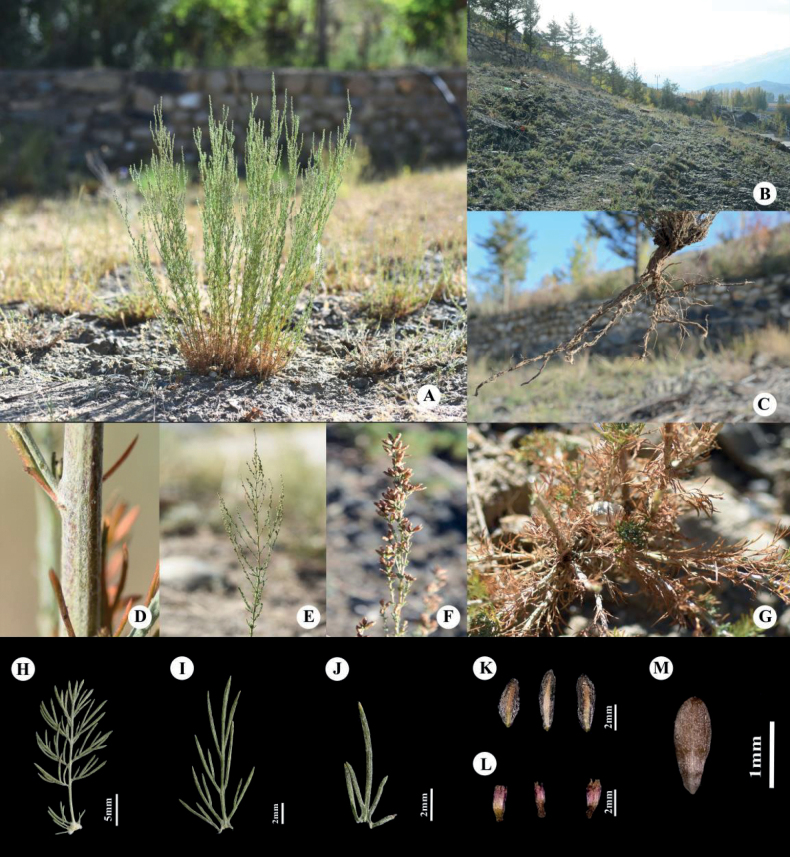
*Artemisiaqingheensis* G. Z. Jin (photographs of the type collection) **A** habit **B** habitat **C** roots **D** stem indumentum **E** compound inflorescence **F** capitula **G** all leaves hardening when mature **H** lower stem leaf **I** middle stem leaf **J** upper leaf **K** involucral bracts **L** florets **M** achene.

**Figure 3.**
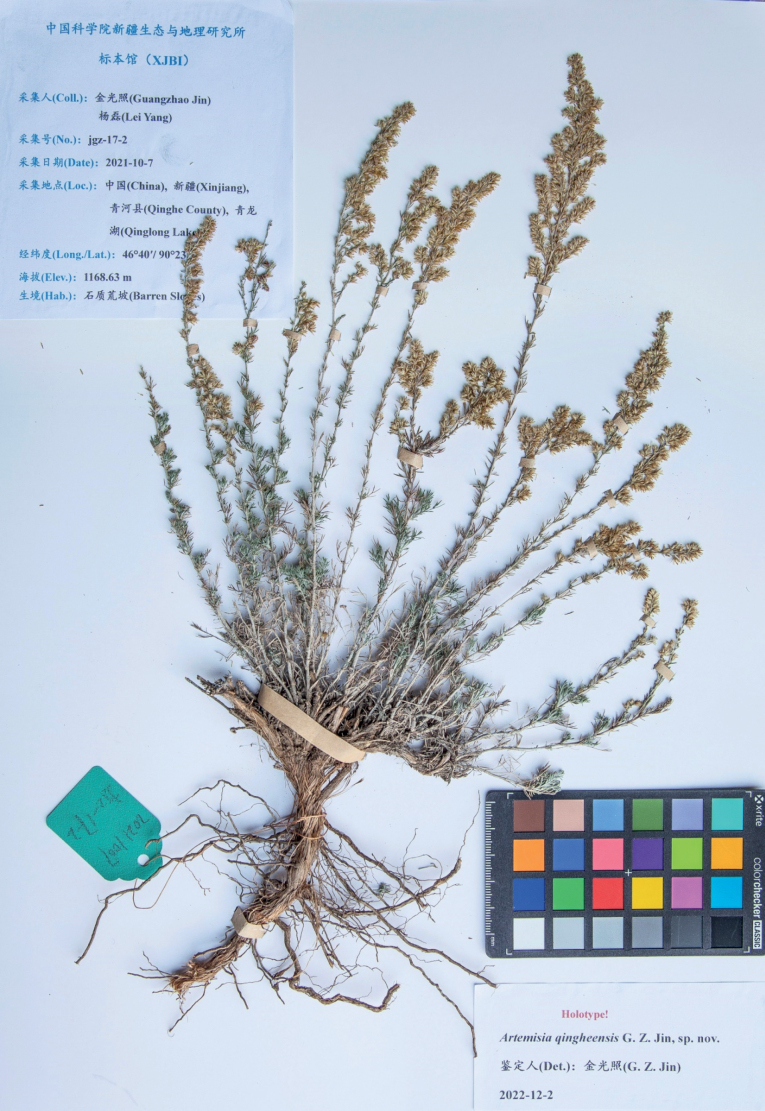
Holotype sheet of *Artemisiaqingheensis* sp. nov.

##### Distribution and habitat.

*Artemisiaqingheensis* is currently only known from Qinghe County, Xinjiang Province, China. It grows on barren slopes at altitudes of 1000 ~ 1500 m.

##### Etymology.

*Artemisiaqingheensis* is named after its type locality, Qinghe County, Xinjiang Province, China.

##### Phenology.

Flowering and fruiting from early September to late October.

##### Vernacular name.

青河绢蒿 (Chinese pinyin: qīng hé juàn hāo). This name is derived from the Chinese name of the type locality.

##### Conservation status.

Although field surveys have been conducted in the north-eastern region of the Junggar Basin over a period of three years, we have only discovered three populations of *Artemisiaqingheensis* in Qinghe County. Unfortunately, as these populations are next to roads and agricultural land, habitat quality is continuously declining due to man-made interference (e.g. grazing, cultivation and landscape engineering). The possible deterioration of its habitat and the restricted distribution of this species threaten its survival. According to the Guidelines for using the IUCN Red List Categories and Criteria (IUCN 2022), the conservation status of *A.qingheensis* should be assessed as Critically Endangered (CR, B1ab).

##### Phylogenetic position and similar species.

**Figure 4.**
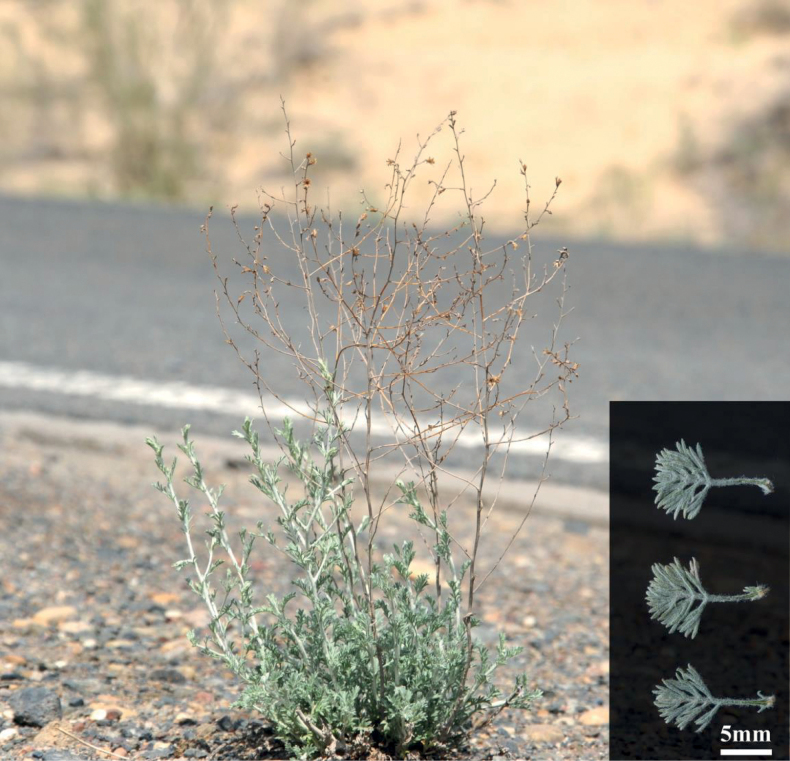
*Artemisiaterrae-albae* (voucher specimen: China. Xinjiang: Mongolian Autonomous County of Hoboksar, 379.32 m alt., 8 May 2022, *Guangzhao Jin 20220508*, XJBI). Inset: Lower stem leaves.

*Artemisiaqingheensis* belongs to Artemisiasubg.Seriphidium because its involucrum is multi-layered, its capitula are homogamous and contain 3–6 bisexual flowers, and these open centrifugally. In addition, our phylogenetic analysis confirmed the inclusion of this new species in subg. Seriphidium. *Artemisiaqingheensis* is similar to *A.terrae-albae* in its habit, leaf shape, petiole length, capitula shape and corolla colour. However, it can be clearly distinguished from *A.terrae-albae* (Fig. 4) because its branches grow adnate to the stem (vs. obliquely upward or spreading) and its leaves harden when maturing (vs. leaves slightly soft when mature). This new species is also relatively easy to distinguish from *A.lessingiana* by its shorter petioles 0.3–1 cm (vs. 2–5 cm) and ovate (vs. oblong-ovate) leaf blade.

The new species is similar to *A.gracilescens* in its habit and narrowly spicate or spicate-paniculate inflorescences. However, it is mainly distinguished from *A.gracilescens* by its 2-pinnatisect lowermost leaves and ovate leaf blade (vs. 2- or 3-pinnatisect and leaf blade triangular-ovate), middle stem leaves 1-pinnatisect (vs. usually 1- or 2-pinnatisect), uppermost leaves three-lobed or undivided (vs. 1- or 2-pinnatisect), all leaves hardening when maturing (vs. leaves slightly soft when mature) and ovoid capitula (vs. ellipsoid). Furthermore, this species is also somewhat similar to *A.amoena* Poljakov in its habit and capitula, which are borne in spikes or narrow panicles, but is distinguished by its shorter petioles 0.3–1 cm (vs. 4–8 cm), longer stem branches: 3–15 cm vs. 2–3 cm, and the hardening of the leaves when these mature (vs. leaves slightly soft when mature).

The morphological differences among *A.qingheensis*, *A.terrae-albae*, *A.lessingiana*, *A.gracilescens* and *A.amoena* are summarised in Table 2.

**Table 2.** Morphological comparisons between *Artemisiaqingheensis* sp. nov. and morphologically similar species.

Character	*A.qingheensis*	*A.terrae-albae*	*A.lessingiana*	*A.gracilescens*	*A.amoena*
Stem	10–40 cm	15–30 cm	18–40 cm	15–30 cm	10–28 cm
Branch	3–15 cm; growing adnate to the stem	3–5 cm; obliquely upward or spreading	3–10 cm; growing adnate to the stem	3–10 cm; growing adnate to the stem	2–3 cm; growing adnate to the stem
Leaf texture	leaves hardening when mature	leaves slightly soft when mature	leaves slightly hardening when mature	leaves slightly soft when mature	leaves slightly soft when mature
Lower leaf	petiole: 0.3–1 cm; leaf blade elliptic, 2-pinnatisect; lobes 2–4 pairs;	petiole: 0.3–1 cm; leaf blade ovate; 1- or 2-pinnatisect; lobes 3–4 pairs	petiole: 2–5 cm; leaf blade oblong-ovate, 1- or 2-pinnatisect; lobes 3–5 pairs	petiole: 0.3–0.5 cm; leaf blade triangular-ovate, 2- or 3-pinnatisect; lobes 2–3 pairs	petiole: 4–8 cm; leaf blade ovate, 1- or 2-pinnatisect; lobes 3–5 pairs
Middle stem leaf	1-pinnatisect	1-pinnatisect	1- or 2-pinnatisect	1- or 2-pinnatisect	1-pinnatisect
Uppermost leaf	three-lobed or undivided	1-pinnatisect	undivided	1- or 2-pinnatisect	undivided
Capitula	ovoid	ovoid	ellipsoidal-ovoid	ellipsoidal	ovoid
Florets	3–6	4–5	5–6	2–5	4–5
Corolla colour	purple-red or yellow	purple-red or yellow	purple-red or yellow	yellow	purple-red or yellow

##### Additional specimens examined

**(paratypes).** CHINA. Xinjiang: Qinghe County, Wolf Garden, 1184.85 m alt., 15 October 2020, *Guangzhao Jin & Sheng Zhang jgz-099* (XJBI); Southern suburb of Qinghe County, 1116.96 m alt., 9 October 2021, *Guangzhao Jin & Lei Yang jgz-25* (XJBI).

### ﻿Key to *Artemisiaqingheensis* and similar species

**Table:** 

1	Petiole of the lower leaves 0.3–1 cm long	**2**
–	Petiole of the lower leaves 2–8 cm long	**3**
2	Branches obliquely upward or spreading, lower leaf 1- or 2-pinnatisect	***A.terrae-albae***
–	Branches growing adnate to the stem, lower leaf 2- or 3-pinnatisect	**4**
3	Lower leaf blade oblong-ovate and capitula ellipsoidal-ovoid, branch length 3–10 cm	***A.lessingiana***
–	Lower leaf blade ovate and capitula ovoid, branch length 2–3 cm	***A.amoena***
4	Leaves harden when maturing, lower leaf blade elliptic	***A.qingheensis***
–	Leaves slightly soft when mature, l ower leaf blade triangular-ovate	***A.gracilescens***

## Supplementary Material

XML Treatment for Artemisia qingheensis

## ﻿Additional information

### Author ORCIDs

Guang-Zhao Jin https://orcid.org/0000-0002-2457-2711

Mariya Sheludyakova https://orcid.org/0000-0001-9504-6033

Wen-Jun Li https://orcid.org/0000-0002-2932-0783

Feng Song https://orcid.org/0000-0002-1332-312X

Zhi-Bin Wen https://orcid.org/0000-0003-1727-2996

Ying Feng https://orcid.org/0000-0002-0817-6751

### Data availability

All of the data that support the findings of this study are available in the main text.
